# A comparison of internal validation techniques for multifactor dimensionality reduction

**DOI:** 10.1186/1471-2105-11-394

**Published:** 2010-07-22

**Authors:** Stacey J Winham, Andrew J Slater, Alison A Motsinger-Reif

**Affiliations:** 1 Department of Statistics, North Carolina State University, Raleigh, NC 27695, USA; 2 Bioinformatics Research Center, North Carolina State University, Raleigh, NC 27695, USA; 3 Department of Genetics, North Carolina State University, Raleigh, NC 27695, USA

## Abstract

**Background:**

It is hypothesized that common, complex diseases may be due to complex interactions between genetic and environmental factors, which are difficult to detect in high-dimensional data using traditional statistical approaches. Multifactor Dimensionality Reduction (MDR) is the most commonly used data-mining method to detect epistatic interactions. In all data-mining methods, it is important to consider internal validation procedures to obtain prediction estimates to prevent model over-fitting and reduce potential false positive findings. Currently, MDR utilizes cross-validation for internal validation. In this study, we incorporate the use of a three-way split (3WS) of the data in combination with a post-hoc pruning procedure as an alternative to cross-validation for internal model validation to reduce computation time without impairing performance. We compare the power to detect true disease causing loci using MDR with both 5- and 10-fold cross-validation to MDR with 3WS for a range of single-locus and epistatic disease models. Additionally, we analyze a dataset in HIV immunogenetics to demonstrate the results of the two strategies on real data.

**Results:**

MDR with 3WS is computationally approximately five times faster than 5-fold cross-validation. The power to find the exact true disease loci without detecting false positive loci is higher with 5-fold cross-validation than with 3WS before pruning. However, the power to find the true disease causing loci in addition to false positive loci is equivalent to the 3WS. With the incorporation of a pruning procedure after the 3WS, the power of the 3WS approach to detect only the exact disease loci is equivalent to that of MDR with cross-validation. In the real data application, the cross-validation and 3WS analyses indicate the same two-locus model.

**Conclusions:**

Our results reveal that the performance of the two internal validation methods is equivalent with the use of pruning procedures. The specific pruning procedure should be chosen understanding the trade-off between identifying all relevant genetic effects but including false positives and missing important genetic factors. This implies 3WS may be a powerful and computationally efficient approach to screen for epistatic effects, and could be used to identify candidate interactions in large-scale genetic studies.

## Background

The identification of genetic factors underlying common, complex diseases such as heart disease or Type II diabetes is a central goal of human genetics. Unlike rare diseases, which often follow simple Mendelian patterns with few genetic variants, these multifaceted diseases are thought to exhibit much more complex genetic etiology, such as interactions between a number of genetic as well as environmental factors [1,2]. Therefore, to fully characterize the genetic architecture of these common complex diseases, we need to consider epistasis, or gene-gene interaction. However, epistasis has proven to be a difficult genetic mechanism to identify with traditional statistical methods, especially as genotyping technology improves and the dimensionality of the data increases [3,4]. Due to the large-scale nature of genetic data (in terms of the number of markers evaluated), we need methods to simultaneously build disease models, perform variable selection, and control for possible false positive findings, which all become inherently more difficult when high-order genetic interactions are considered [2,5,6].

In order to tackle this problem, a variety of novel data-mining methods have been developed. Multifactor Dimensionality Reduction (MDR) [7] is one such method that evaluates potential interactions by performing an exhaustive search of all variables and variable combinations through attribute construction to collapse multi-locus genotype combinations into high-risk and low-risk categories. Much work has been done on MDR and many extensions have been developed [8-15]. It is arguably one of the most commonly used data-mining methods in genetic epidemiology [7,16], and has been highly successful in a wide range of simulations [6,17-20] and real data applications, including investigations of multiple sclerosis [21,22], schizophrenia [23], and endophenotypes in breast cancer [24].

However, there are still a number of limitations of the method, one of which is computation time due to its combinatorial, exhaustive search nature. An important issue in general data-mining is model over-fitting, where models are trained too closely on limited available data and do not generalize well to new unseen data [25]. Since the overall goal of association mapping is to detect genetic associations that generalize to whole populations, traditionally the "gold standard" in evaluating a true association is through replication [26]. But because study replication is an ideal standard and not always a reality, we need to reduce false positive results within a single study. In order to lessen the potential for false positives, an identified model needs to not only fit the sample data well, but also needs to be a good predictor of disease status in the population. Thus estimates of prediction error from the sample data are paramount, and are achieved through methods of internal validation. MDR currently relies on cross-validation for internal validation. Cross-validation has been proven successful in detection of interactions in a variety of studies, but it is computationally expensive, particularly for an exhaustive search technique like MDR [7,22]. It is possible that by considering alternatives to cross-validation, we could improve the speed and performance of the method.

A commonly used internal model validation method in data-mining is a three-way split of the sample data, as an alternative to cross-validation [25]. We will refer to this as simply three-way split (3WS), but it should not be confused with the three-way split for decision trees [25]. For this type of internal model validation, the original data is split into a training set for model building, a testing set for refining, and a validation set to assess predictive capability, resulting in lower total number of repetitions of the algorithm and much lower computation time as compared to cross-validation. Another advantage of the method is the two-stage model-building procedure prior to validation; only models from the training set which replicated in the testing set are considered for validation, which provides evidence of replication without collecting a new sample. Based on these potential advantages, we have incorporated the 3WS internal validation scheme into the MDR method; as well as post-hoc pruning procedures to potentially further reduce false positives. While the 3WS can dramatically reduce computation time, it is unknown how it will affect the power of MDR to detect true disease causing loci, particularly for candidate gene studies in case/control data. In order to investigate this, we designed a Monte Carlo simulation study to compare the power of the traditional MDR method with cross-validation to MDR using 3WS with and without post-hoc pruning. The goal of this study is to determine whether what is gained in computation time for the 3WS is lost in terms of power to identify genetic variants of common, complex disease. We evaluate the relative performance of MDR using the 3WS (both with and without pruning) to both 5-fold and 10-fold cross-validation. We compare the power of the method to detect disease causing models, the bias and variance of prediction error estimates, and computation time using both internal validation techniques. Additionally, we evaluate a range of parameter settings related to the 3WS, including different proportions of the data for each split and the number of models passed through the splits, to optimize the approach. We also investigate a range of options for the post-hoc pruning procedure and demonstrate the relative advantages and disadvantages of each. Finally, we illustrate the effectiveness of 3WS with a real data example involving CD4 immune recovery in response to therapy for Human Immunodeficiency Virus-1 (HIV-1) patients, identifying a two-locus interaction that predicts immune response.

## Methods

### Statistical Methods

#### Multifactor Dimensionality Reduction

Multifactor Dimensionality Reduction is a data-mining method utilizing combinatorial data reduction techniques to accommodate gene-gene and gene-environment interactions [7]. MDR is nonparametric in both the statistical and genetic sense, as no assumptions are made concerning statistical distributions or genetic models [8]. To illustrate the method, suppose we have a total sample size *n *with *n*_*1 *_cases and *n*_*0 *_controls. Additionally, suppose we have *K *total loci and we are considering *k *loci for interaction. With *k *loci considered for interaction and 3 genotypes per locus, the data can be classified into 3^*k *^possible genotypic combinations. MDR reduces these combinations by calculating the ratio of cases to controls within each of the 3^*k *^multi-factorial classes, and then labeling the class (i.e. genotypic combination) as either "high-risk" or "low-risk" based on this ratio exceeding a given threshold, such as *n*_*1*_/*n*_*0 *_(1.0 in the case of balanced data). Therefore, MDR reduces the *k*-dimensional space to one-dimension with two levels ("high-risk" and "low-risk"), and this high-risk/low-risk parameterization of genotype combinations comprises the MDR model for the particular loci involved.

Let model *i *represent an arbitrary combination of *k *loci. Each possible model *i *would classify an individual as a case if that individual's genotype combination at the *k *loci were characterized as high-risk. Intuitively, in order to select a final model we would like to minimize misclassification or equivalently maximize a measure of classification accuracy. Let *n*_*11,i *_be the number of true cases who were correctly classified as cases and let *n*_*00,i *_be the number of true controls correctly classified as controls by model *i. *Now we can define balanced accuracy for model *i *as *BA*_*i*_, the arithmetic mean of sensitivity and specificity, where

**Figure 1:**
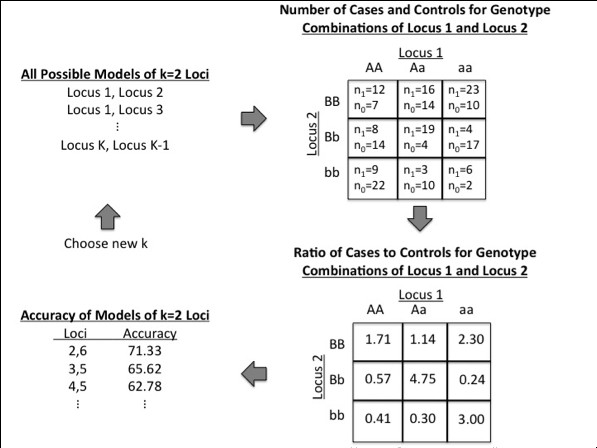
**Overview of the MDR method for *k *= 2 loci**. First all possible combinations of *k *= 2 loci are enumerated. For a given combination of loci, the number of cases and controls are tabulated for each genotype combination, and then the ratio of cases to controls is calculated within each cell. If the ratio exceeds a threshold (1.0 here) then the combination is labeled as high risk, otherwise it is labeled as low risk. This high-risk/low-risk characterization is the MDR model for that combination of loci, and the accuracy of the model is determined. The model that maximizes accuracy is chosen as the best model of size k. Repeat the process for a new *k*.

For balanced studies with an equal number of cases and controls, balanced accuracy is equivalent to classification accuracy, the proportion of correctly classified individuals [20]. MDR will select the combination *i *of *k *loci which will maximize the balanced accuracy, *BA*_*i*_, (or equivalently minimize the balanced error *BE*_*i *_= 1-*BA*_*i*_) based on the high-risk/low-risk parameterization. This combination of loci will be the best model for a *k*-factor interaction. A final best model over all possible sizes of interaction is chosen with an internal validation procedure, such as cross-validation or a three-way split. The statistical significance of the estimate of balanced accuracy/error from the final model can be evaluated through permutation testing. An overview of the general procedure for models of size *k *can be seen in Figure 1.

#### MDR with *m*-fold Cross Validation

**Figure 2:**
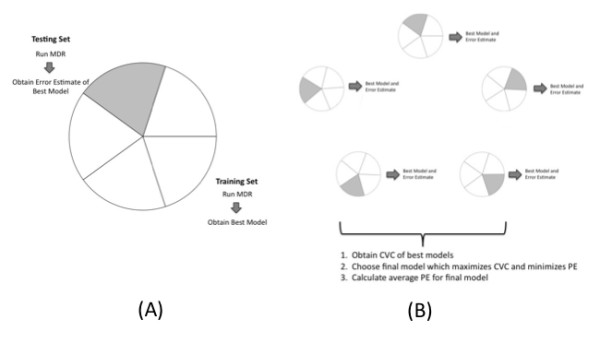
**Cross Validation Approach. **A) 5-fold cross-validation split of the full sample data. B) Explanation of how 5-fold cross-validation is incorporated into the MDR method. First the sample data is randomly split into 5 intervals with representative numbers of cases and controls in each interval. MDR is performed on each of the 5 possible splits. Cross-validation consistency (CVC) is calculated for each of the best models and a final model is chosen which maximizes CVC and minimizes prediction error (PE).

After a best model is determined for every possible model size *k *of interest, an overall best model is selected based on predictive capability, which is traditionally assessed using *m*-fold cross-validation. Prior to any data reduction, the complete data is separated into *m *equal intervals. The training set is made up of *m-1 *intervals and the testing set is the remaining interval (Figure 2a). The best MDR model for *k *loci is determined from the training set and an estimate of the model's prediction accuracy *PA*_*i *_is calculated, where prediction accuracy is classification accuracy calculated from the testing set rather than the training set. This procedure is repeated for all *m *possible splits of the data (Figure 2b). Cross-validation consistency is then determined for each of the "best models", where cross-validation consistency is defined as the number of times a particular model is identified across all *m *cross-validation subsets [27]. The final model will then be chosen as that which maximizes both prediction accuracy (or minimizes prediction error) and cross-validation consistency over the set of "best models"; if the model which maximizes prediction accuracy differs from the model which maximizes cross-validation consistency, the more parsimonious model is chosen [8]. The average prediction accuracy/error of this final model is the measure of predictive capability. MDR with cross-validation is outlined in more detail in [28]. We utilize both 5-fold and 10-fold cross-validation in this study.

#### MDR with Three-Way Split

**Figure 3:**
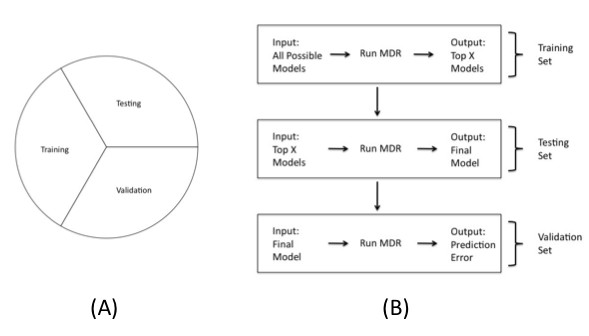
**Three Way Split Approach.** A) Three-way split of the full sample data. B) - Explanation of how the three-way split is incorporated into the MDR method. First the sample data is randomly split into 3 intervals with representative numbers of cases and controls in each interval. MDR is performed on each of the three splits with all possible models considered in the training set, the top *x *models considered in the testing set, and the final model considered in the validation set for each *k*.

Prior to data reduction, the full data set is randomly split into three pieces: a training set for model building, a testing set for refining, and a validation set to assess predictive capability (Figure 3a). The three splits of the data can be thought of as independent replication sets, where the first set is used to identify plausible models, the second set is used to determine whether these models replicate, and the third set is used to validate the results by obtaining prediction estimates. MDR is performed in each of the three sets, with the largest number of models being considered in the training set, a reduced number of models considered in the testing set, and a small number of models considered in the validation set. For each combination of loci considered in each of the three stages, the high-risk/low-risk MDR parameterization and the resulting balanced accuracy is determined.

MDR will be implemented first in the training set for all possible combinations of loci for each model size *k *of interest. For each model size *k*, the models considered will be ranked in terms of balanced accuracy and the *x *models with the highest balanced accuracy for each *k *will be preserved for evaluation in the testing set.

These top *x *models for each size of interaction will then be considered in the testing set. In the testing set, MDR will be performed on all *x *models preserved from the training set. The models will be ranked in terms of balanced accuracy and the best model will be retained for evaluation of predictive capability in the validation set. This process is repeated for each *k*.

The single top model for each size *k *from the testing set will be considered in the validation set. MDR will be performed on all of the top models for the data in the validation set, and the balanced accuracies from the validation set will be retained. A final model will be chosen as the model that maximizes the balanced accuracy in the validation set among all top models for the interaction sizes considered. This maximum balanced accuracy (or minimum balanced error) will be the measure of predictive ability of the final model. The main steps of the three-way split method for an interaction of size *k *are outlined in Figure 3b.

In this initial implementation, we utilize three equal splits of the data and a value *x *equal to the total number of loci, but in realizing that these parameter settings are arbitrary, we perform parameter sweep experiments to find more optimal values. We investigate different options for the proportion of data in each subset as well as different values for the threshold *x *in order to optimize the performance of 3WS with MDR with respect to power and provide users with guidance for setting these parameters.

#### Post-hoc pruning

Because BA will increase for larger orders of interaction, MDR will tend to select larger models. This problem is alleviated in cross-validation through the use of CVC and the parsimony rule, but there is no analogous mechanism for 3WS. To address this, after implementation of MDR with 3WS, we also assess a number of possible post-hoc pruning procedures based on logistic regression (on the entire dataset) to further reduce potential false positives and to provide a mechanism to obtain more parsimonious models. Many other options for post-hoc pruning are possible, and logistic regression is simply one possibility that is widely recognized and available in most software packages. After a final model has been determined with MDR using 3WS, we evaluate the impact of pruning back the total number of identified loci using backward model selection with logistic regression. To keep with the nonparametric nature of the MDR method, the logistic regression model utilizes genotype indicator variables to avoid making any assumptions about the genetic mode of inheritance, and all possible interactions between identified loci are considered for the full model. Backward selection can then be performed using either a pre-specified p-value threshold or by minimizing an information criterion, such as AIC or BIC, to sequentially remove variables from the model. The post-pruning model is defined as the loci involved in the remaining variables after backward selection. We evaluate a range of analysis choices for this pruning procedure, and the relative performance of each for maximizing power.

#### Simulation Design

In order to compare the internal validation techniques of cross-validation and the three-way split within the MDR method, we performed a Monte Carlo simulation study. Factors of interest were chosen as number of loci involved in the true disease model, minor allele frequency (MAF), and effect size characterized in terms of heritability (h^2^) and odds ratio (OR). We considered true disease models of size one, two, three, and four loci. Models of size one are main effects models with dominant, recessive, and additive genetic effects. In contrast, all models of size two and greater are models with epistatic interactions; these epistatic models exhibit marginal to no main effects. Minor allele frequencies considered for each model were 0.25 and 0.5 to represent relatively common variants. Heritabilities considered were 0.01, 0.05 and 0.10 to represent disease models with relatively low genetic signals. Odds ratios considered ranged from 1.25 to 5.1, where the lower odds ratios correspond to h^2 ^= 0.01 and the higher odds ratios correspond to h^2 ^= 0.10. For disease models with binary outcomes, heritability and the odds ratio are both calculated based on the probability of developing disease given an individual's genotypic information, therefore, there is an algebraic relationship between them; lower values of heritability must be considered for lower odds ratios. Heritabilities were calculated as previously described [29].

The effect sizes exhibited here were chosen to have low genetic signals. For instance, Alzheimer's disease is estimated to have heritability between 0.40 and 0.70 [30]. By considering models with low genetic signals, we are able to better compare the two internal validation techniques at the lower limits of power. Implicitly, we assume if a method is able to detect a small effect, it should have high power to detect large effects. For the single locus models, all combinations of the three genetic models (additive, dominant, and recessive effects), the two minor allele frequencies, and two values of heritability (0.01, 0.05) with ORs of 1.5 and 2.5 respectively were simulated, resulting in a total of 12 single locus models. For the two-locus epistatic models, all combinations of the two minor allele frequencies and three values of heritability (0.01, 0.05, and 0.10) with a range of ORs for each value of heritability were generated, resulting in 22 total two-locus models. For the three-locus epistatic models, all combinations of the two minor allele frequencies and three values of heritability (0.01, 0.05, and 0.10) with a range of ORs for each heritability value were generated, resulting in 20 total three-locus models. For the four-locus epistatic models, all combinations of the two minor allele frequencies and three values of heritability (0.05, 0.10, and 0.15) with two ORs for each heritability value were generated, resulting in 12 total four-locus models. These parameter choices result in a total of 66 combinations that are listed in detail in Additional File 1: Supplemental Table S1.

#### Data Generation

For each of the 66 combinations of factors, we generated 100 Monte Carlo datasets under a balanced case-control setting, designed to reflect an epidemiological candidate gene study. A total sample size of 1000 (500 cases and 500 controls) was utilized and 25 independent loci were generated for each individual assuming Hardy-Weinberg Equilibrium. To reduce the computational burden of this large-scale simulation, only 25 loci were generated, which is much smaller than what we could expect to see in a typical candidate gene study. It has been previously shown that additional nuisance loci do not affect the power of the MDR method and therefore our results should appropriately scale up to larger studies [17]; however, to validate this claim, we also compared the results for selected models generated with 100 total loci.

Case-control data were generated using penetrance functions, where penetrance is defined as the probability of disease given the genotype at the disease locus. For epistatic models, the penetrance is the probability of disease given the combination of genotypes at the disease loci. In addition to the epistatic models previously described, we also included two commonly studied epistatic models for two-locus interactions, XOR [31] and ZZ [32], which are well-described theoretical examples of epistasis with no main effects at either locus. The penetrance functions for these models are depicted in Table 1. For example, under the XOR model, an individual with genotype AABB at the two disease loci incurs no disease risk, while an individual with genotype AABb has a 10% risk of disease. For the special cases of the XOR, ZZ, dominant, additive, and recessive models, penetrance functions were explicitly determined. For all other epistatic models, penetrance functions were generated with an evolutionary computation algorithm, SimPen [33], to achieve the desired minor allele frequency, heritability, and odds ratio as well as to minimize the marginal effects at each individual disease locus, and are available upon request. These penetrance functions were then utilized to generate 100 datasets for each model using the software genomeSIM [34].

**Table 1:** Penetrance function for the XOR and ZZ models

Model	XOR			ZZ		
**Genotype**	**AA**	**Aa**	**aa**	**AA**	**Aa**	**aa**

**BB**	0.0	0.1	0.0	0.0	0.0	0.1

**Bb**	0.1	0.0	0.1	0.0	0.05	0.0

**bb**	0.0	0.1	0.0	0.1	0.0	0.0

Penetrance is the probability of developing disease given an individual's genotype combination at Locus A and Locus B.

#### HIV Immunogenetics Data

In the present study, HIV immunogenomics data is used not to discover new genetic associations, but to evaluate potential differences in the results of MDR with cross-validation and the 3WS plus pruning strategies. Additionally, we use the real data application to demonstrate the parameter settings indicated for optimizing power in the simulation studies. Details of the study and dataset have previously been described in detail [35]. The phenotype of interest in this study was CD4 cell increase in n = 873 HIV patients initiating potent antiretroviral therapy, measured as the CD4 cells/mm^3 ^increase from pre-treatment baseline to 48 weeks of virologic control on treatment for each patient. The outcome was dichotomized at ≥200 CD4 cells/mm^3 ^and < 200 CD4 cells/mm^3^, representing "immune response" and "non-response" categories, respectively. A total of 35 SNPs in genes that encode proteins that play an important role in interleukin-2/interleukin-15 signaling were genotyped and MDR (with 5-fold cross-validation) was used to evaluate potential gene-gene interactions. Results indicated a two-locus interaction between polymorphisms in genes encoding IL-2Rβ (16491C > G) and IL-2Rγ (4735T > C) (also known as CD132) that predicted response with 57% accuracy [35].

#### Analysis of Simulated Data

All 100 datasets for all 66 considered models were analyzed with MDR using both 5-fold and 10-fold cross-validation (CV-5 and CV-10) and MDR with 3WS before pruning, using three equal splits of the data. For the analysis using 3WS, *x *= 25 (the number of total loci) top models in the training set were preserved for evaluation in the testing set. For both methods, MDR considered model sizes of *k *= 1,...,3 loci for true one- and two-locus models and model sizes of *k *= 1,...,4 loci for true three- and four-locus models.

For each of the 66 models, power to detect the true simulated disease loci was calculated across all 100 datasets as the proportion of times a correct model was identified. Two definitions of power were considered: conservative and liberal. Under the conservative definition, a model was correct if and only if all of the true disease loci were identified exactly, with no false positive loci present. Under the liberal definition, a correct model was only required to contain the true disease loci, allowing for possible false positives but not false negatives. We consider false positive rather than false negative loci because we implicitly assume that failing to discover important functional loci is a more critical error than including non-functional loci. To illustrate the difference between these two definitions, consider a true simulated disease model involving locus 1 and locus 2; an MDR result which identified loci 1, 2, and 3 would be correct under the liberal definition but not under the conservative definition. Conservative power measures the ability of a method to discard false positive loci while still retaining true associated loci, while liberal power measures a method's utility as a screening tool which will not miss important genetic factors. Methods with high conservative power have both high sensitivity and specificity, while methods with high liberal power exhibit high sensitivity only. Because only models of up to *k *= 4 loci were considered, for true models of size four, only the conservative power definition was relevant; since MDR could identify no more than four loci, it would be impossible for it to identify all four simulated loci plus false positives and therefore, liberal and conservative power are equivalent.

It has been previously shown that the use of 5-fold and 10-fold cross-validation lead to similar results for MDR [18], but we compare the performance of CV-5 and CV-10 for all 66 models to validate this assumption for our study. It has also been previously shown that increasing the number of nuisance loci does not affect the power of MDR to detect the true disease causing loci [17], but we also compare the results of CV-5, CV-10, and 3WS for both the XOR and ZZ models using data generated with 100 total loci in addition to 25 loci. We also consider the use of a two-way split (2WS) of the sample data, with equal size training and testing sets and no validation set, for comparison to 3WS for the XOR and ZZ models.

Although the focus of this study is on power to detect a true disease model, the prediction accuracy of each internal validation method was also evaluated in terms of prediction error. Bias and variance of the estimates of prediction error for both CV-5 and 3WS internal validation methods were calculated for each model as the Monte Carlo average across the 100 datasets.

All simulation results were statistically evaluated under a general linear mixed model framework, treating each combination of factors as a single observation and the final results for power, bias, and variance as response variables. Four separate linear mixed effects models were fit to the response variables of conservative power, liberal power, bias, and variance, respectively. Minor allele frequency, heritability, odds ratio, size of true, simulated interaction, and internal validation method were treated as fixed explanatory variables. For a given simulated model (i.e. combination of simulation factors), both internal validation methods were performed on the same 100 datasets, and therefore the CV and 3WS estimates can be viewed as repeated measurements on a particular simulated model. Because the same datasets for a given simulated model are utilized, the estimates produced from both CV and 3WS will be correlated. A random effect for each simulated model was included to account for this dependence. This type of mixed-effects analysis approach is common for repeated measures data, and for more information see [36,37]. The results from this analysis allow us to determine which factors greatly influence power, and prediction bias and variance, and allow us to statistically determine whether or not the two internal validation methods of MDR lead to differing results.

Initial comparisons were performed using 3WS with three equal splits of the data (1:1:1) and a threshold value of *x *= 25, but we realize that these may not be the optimal parameter settings in terms of power. For a subset of the original 66 models involving both two- and three-locus interactions, we investigate MDR with 3WS using seven different proportions of data in each of the training, testing, and validation subsets (1:1:1, 1:1:2, 1:2:1, 2:1:1, 2:2:1, 2:1:2, and 1:2:2) and four possible values of *x *(15, 25, 50, and 100) using a two-stage analysis approach. In the first stage, five of the 22 two-locus models were selected for a range of effect sizes at minor allele frequency of 0.5. All 100 datasets for each model were re-analyzed for each of the 28 combinations of *x *and data proportion. Conservative and liberal power were calculated for 3WS under each of these parameter combinations and we determined a set of potential optimal values for these parameters by maximizing liberal power; we utilize liberal power to maximize the number of true positives identified without concern for false positives, since we will ultimately incorporate a post-hoc procedure to prune back the false positives. In the second stage, we identify a single optimal value for both *x *and the data proportions from the set of potential optimal values identified in the first stage by incorporating four of the 20 three-locus models (also selected for a range of effect sizes at minor allele frequency of 0.5) into the comparison. Conservative and liberal powers were compared for the nine two-locus and three-locus models considered and a final optimal value of *x *and the data proportions were chosen. We analyze the results using a mixed effects model with a random effect for each of the considered simulated models, and compare potential parameter combinations using pair-wise contrasts.

After optimization of the parameters for 3WS, pruning was incorporated using logistic regression with various selection criteria, including AIC, BIC, and p = 0.1, 0.05, 0.01, 0.001, 1E-4, 1E-5, 1E-6, and 1E-7. The optimal data proportions and threshold *x *were used to compare both liberal and conservative power for each of the selection criteria, 3WS without pruning, and CV-5. To determine if pruning could improve conservative power without adversely affecting liberal power, since our goal is to reduce the number of false positives identified without reducing true positives, these results were also analyzed with a mixed effects model.

All data analysis was performed in R software [38] on the High Performance Computing cluster resource (http://hpc.ncsu.edu), or using SASv9.1.3 (http://www.sas.com). Code to implement both MDR with cross-validation and MDR with a three-way split (both with and without pruning) are available from the authors upon request.

## Results

### Simulation Results

For all 66 models, results for both conservative and liberal power are similar for MDR with 5-fold and 10-fold cross-validation, confirming our assumptions (p = 0.10; see Additional File 2: Supplemental Table S2). Additionally, for both the XOR and ZZ models, results for CV-10, CV-5, and 3WS are similar when the number of total loci was increased from 25 to 100, which is consistent with the assumption that the addition of nuisance loci does not affect the power of MDR (see Additional File 3: Supplemental Table S3). Because both of these assumptions have been validated, the effect of the number of cross-validation intervals and nuisance loci will no longer be discussed in the current manuscript. Power results for MDR with 3WS and 2WS for a small subset of models are also similar, indicating that perhaps 2WS might be sufficient, although this needs further investigation (see Additional File 4: Supplemental Table S4).

**Figure 4:**
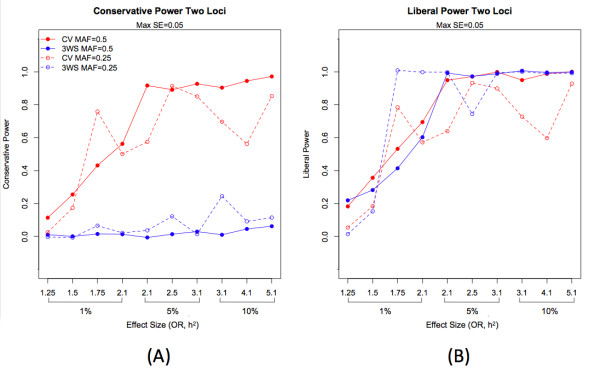
**Conservative (A) and liberal power (B) for epistatic models with two loci**. Conservative and liberal power is plotted for increasing effect size for both MDR with a three-way split (3WS) and cross-validation (CV). Effect size is ordered first in terms of heritability and within a single level of heritability then in terms of odds ratio. Maximum standard errors for power estimates are 0.050 for both conservative and liberal power.

**Figure 5:**
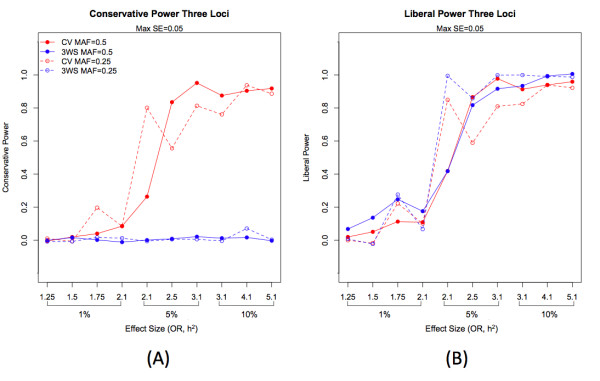
**Conservative (A) and liberal power (B) for epistatic models with three loci**. Conservative and liberal power is plotted for increasing effect size for MDR with 3WS and CV. Maximum standard errors for power estimates are 0.050 for both conservative and liberal power.

For the dominant, recessive, and additive disease models for a single locus, 5-fold cross-validation outperforms the 3WS in terms of both conservative and liberal power (Table 2). However, for the epistatic models of size 2 and 3, the 3WS generally gives similar liberal power, and slightly higher liberal power for models of higher effect size (OR and h^2^) and minor allele frequency of 0.25. Generally, 5-fold cross-validation yielded higher conservative power estimates, particularly for higher effect sizes (Figures 4 and 5).

**Table 2:** Conservative and liberal power results for all single locus models.

				Conservative Power				Liberal Power			
**MAF**	**h**^**2**^	**OR**	**Model Name**	**CV-5**	**SE**	**3WS**	**SE**	**CV-5**	**SE**	**3WS**	**SE**

0.5	0.01	1.5	Dominant	0.52	0.05	0.00	0.00	0.67	0.05	0.59	0.05

0.5	0.01	1.5	Recessive	0.67	0.05	0.00	0.00	0.75	0.04	0.68	0.05

0.5	0.01	1.5	Additive	0.45	0.05	0.00	0.00	0.68	0.05	0.58	0.05

0.5	0.05	2.5	Dominant	0.94	0.02	0.00	0.00	1.00	0.00	1.00	0.00

0.5	0.05	2.5	Recessive	0.93	0.03	0.00	0.00	1.00	0.00	1.00	0.00

0.5	0.05	2.5	Additive	0.91	0.03	0.00	0.00	1.00	0.00	0.93	0.03

0.25	0.01	1.5	Dominant	0.77	0.04	0.01	0.01	0.83	0.04	0.54	0.05

0.25	0.01	1.5	Recessive	0.11	0.03	0.00	0.00	0.24	0.04	0.14	0.03

0.25	0.01	1.5	Additive	0.68	0.05	0.00	0.00	0.80	0.04	0.36	0.05

0.25	0.05	2.5	Dominant	0.88	0.03	0.00	0.00	1.00	0.00	1.00	0.00

0.25	0.05	2.5	Recessive	0.85	0.04	0.00	0.00	0.96	0.02	0.78	0.04

0.25	0.05	2.5	Additive	0.91	0.03	0.00	0.00	1.00	0.00	1.00	0.00

Power results are shown for both the three-way split and 5-fold cross-validation by mode of inheritance, odds ratio (OR), heritability (h^2^), and minor allele frequency (MAF). Maximum standard errors of all power estimates are also included.

**Figure 6:**
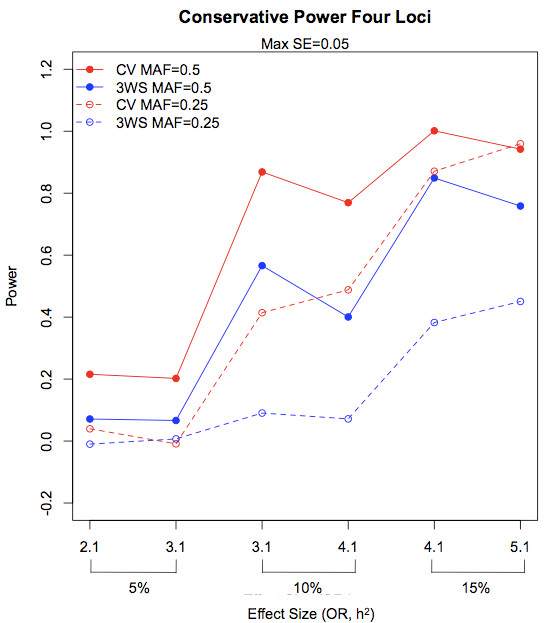
**Conservative power for epistatic models with four loci**. Conservative power is plotted for increasing effect size for MDR with 3WS and CV. Maximum standard errors for power estimates are 0.050.

For four-locus epistatic models (where the search of the model space was restricted to four loci), conservative power was highest for CV-5 (Figure 6). Both conservative and liberal power results across effect size can be seen in Figures 4, 5, 6 for models of size 2, 3, and 4, where effect size refers to the ordering of effect in terms of heritability and odds ratio. Many of the differences seen between the two internal validation methods are greater than 0.05, the maximum standard error of all power estimates, indicating that these differences are not simply due to chance variation; numerical results for each observation as well as standard errors can be found in Additional File 2: Supplemental Table S2. Results for the two-locus interaction XOR and ZZ models are similar to those described above, with higher conservative power for cross-validation but similar liberal power for both methods (Table 3).

**Table 3:** Conservative and liberal power for special epistatic models of size two loci.

			Conservative Power				Liberal Power			
**MAF**	**h**^**2**^	**Model Name**	**CV**	**SE**	**3WS**	**SE**	**CV**	**SE**	**3WS**	**SE**

0.5	0.05	XOR	0.92	0.03	0.39	0.05	1.00	0.00	1.00	0.00

0.5	0.05	ZZ	0.92	0.03	0.70	0.05	0.99	0.01	1.00	0.00

Power results are shown for both the three-way split and 5-fold cross-validation for the XOR and ZZ epistatic models by heritability and minor allele frequency. Maximum standard errors of all power estimates are also included.

Additionally, 3WS tends to choose final models that are as large as allowed by the user before pruning (in this study, either three or four loci), while 5-fold cross-validation trends towards more parsimonious models. For instance, for true models of size three, average model size (number of loci included) was 3.99 (SE = 0.10) for 3WS and 1.71 (SE = 0.96) for CV-5. This trend in average model size is similar for all true sizes, indicating that 3WS before pruning favors false positives, possibly representing a tendency towards over-fitting, while CV-5 favors false negatives (Table 4).

**Table 4:** Average size of selected model by true model size.

	3WS		CV-5	
**True Size**	**Average Size**	**SE**	**Average Size**	**SE**

**1**	2.94	0.008	1.30	0.019

**2**	2.90	0.006	1.91	0.012

**3**	3.99	0.002	2.38	0.020

**4**	3.99	0.003	3.17	0.033

Comparison of average model size for both the three-way split and 5-fold cross-validation. For true sizes of one and two, MDR was performed for one to three loci. For true sizes of three and four, MDR was performed for one to four loci.

As expected, for both methods and both power definitions, power generally increases with effect size (heritability and odds ratio) and with minor allele frequency. The results of the mixed effects model analysis yielded that after accounting for model size, heritability, odds ratio, and minor allele frequency, conservative power is significantly higher for CV-5 than for 3WS (p < 0.0001), but in terms of liberal power, there is no difference between the two methods (p = 0.2784). These results can be seen in full in Table 5. Upon further investigation, possible interactions between the simulation design factors were considered, exposing a potential interaction between model size and type of internal validation method (p < 0.0001 for both conservative and liberal power); while increased model size leads to decreased power for both internal validation methods, the decrease in conservative power is less substantial for the 3WS compared to CV-5 and the decrease in liberal power is more substantial. Additionally, bias of the prediction estimate did not differ between the methods (p = 0.5406) but variance was greater for the three-way split (p < 0.0001; data not shown). This is not surprising, since cross-validation involves an average across all cross-validation intervals to achieve the prediction error of the final model, therefore resulting in a less variable estimator.

**Table 5:** Significance of simulation factors on conservative and liberal power.

Effect	P-value (conservative power)	P-value (liberal power)
Size	0.0043	< 0.0001

MAF	0.1533	0.0850

h^2^	0.0005	0.0003

OR	0.7620	0.0507

Method	<0.0001	0.2784

P-values for model size, minor allele frequency, heritability, odds ratio, and type of internal validation method in terms of conservative and liberal power from the repeated measures analysis of the simulation results comparing CV-5 and 3WS without pruning. P-values less than 0.05 are considered statistically significant.

In terms of the possible values of the threshold parameter *x *for 3WS, the mixed effects model analysis indicated the differing choices of *x *did not lead to significantly different conservative or liberal power in the first stage analysis (p = 0.5547 and p = 0.4333, respectively; see Additional File 5: Supplemental Table S5 and Additional File 6: Supplemental Table S6). Both power measures were maximized for 3WS using *x *= 25, so *x *was fixed at 25 for the second stage analysis. Both conservative and liberal powers were significantly different for the choices of data sub-setting in the first stage (p = 0.0260 and p < 0.0001, respectively; Additional File 6: Supplemental Table S6). Liberal power was maximized for the data proportions 2:2:1, although the subset 2:1:1 was not significantly different (p = 0.0907). The results for the first stage analysis can be seen explicitly in Additional File 5: Supplemental Table S5 and Additional File 6: Supplemental Table S6. In the second stage, the top three proportions (2:2:1, 2:1:1, and 1:2:1) were further tested and the combined results can be seen in Additional File 7: Supplemental Table S7. After accounting for model size and effect size, there was a difference in liberal power between the three proportions (p = 0.0022; see Additional File 8: Supplemental Table S8) with the same 2:2:1 proportion yielding the highest liberal power. Contrasts of power estimates against the other two proportions indicated only the 1:2:1 proportion was different (p = 0.0006; see Additional File 8: Supplemental Table S8).

**Figure 7:**
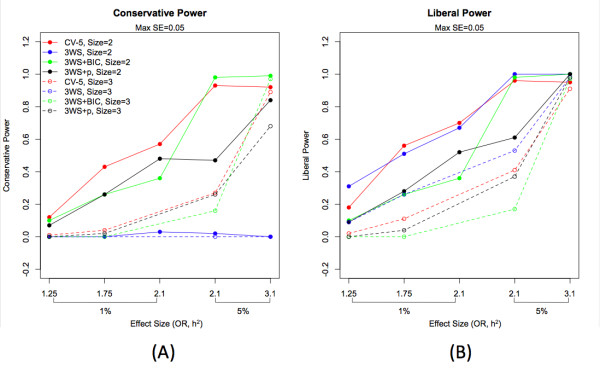
**Conservative (A) and liberal power (B) for epistatic models for two and three loci with MAF = 0.5**. Both conservative and liberal power is plotted for optimized 3WS without pruning, 3WS with BIC, 3WS with p = 0.001, and CV-5 for increasing effect size. Effect size is ordered first in terms of heritability and within a single level of heritability then in terms of odds ratio.

When the post-hoc pruning procedures were incorporated into 3WS with the optimal parameter choices (*x *= 25 and subset = 2:2:1), both conservative and liberal power differed significantly among the pruning methods after controlling for the effects of model size and effect size (p < 0.0001; see Additional File 9: Supplemental Table S9 and Additional File 10: Supplemental Table S10). For conservative power, CV-5 yielded the highest response and contrasts against the power estimates of the pruning approaches showed several to be statistically similar (Additional File 10: Supplemental Table S10). In fact, among the 3WS approaches, conservative power was maximized for selection utilizing BIC and was not significantly different from CV-5 (p = 0.6114), with a significant improvement over the use of 3WS alone (p < 0.0001). Liberal power was maximized for 3WS with no pruning, as expected, with several of the pruning approaches being statistically similar and the smallest loss for the loose p-value threshold of 0.1 (p = 0.8804). In order to increase conservative power without drastically reducing liberal power, pruning with a threshold of *p *= 0.001 is a nice compromise. Both conservative and liberal power results are compared for the optimized 3WS without pruning, 3WS with BIC, 3WS with *p *= 0.001, and CV-5 in Figure 7. All pruning results and contrasts can be seen in Supplemental Tables S9 and S10 (Additional Files 9 and 10).

In terms of computation time, theoretically the three-way split is approximately five times faster than 5-fold cross-validation. The bulk of computing time is spent in exhaustively constructing the MDR classifier and evaluating BA for all possible combinations of loci in the training set, which is performed only a single time for 3WS and 5 times for CV-5; the additional computing time due to the small number of combinations of loci considered in the testing set and validation set of 3WS followed by pruning and the additional computing time due to the testing set in CV is negligible. This approximate 5-fold reduction is theoretical, and the exact reduction in computing time for 3WS from CV-5 will depend on a number of parameters such as the overall sample size, the number of total loci, and the sizes of interaction considered; additionally, the size of the split proportions and the value of the threshold *x *will also effect computation time for 3WS. Specifically, when we consider one to four-way interactions for a single dataset with sample size of 1000 and 25 loci, 3WS before pruning (with split proportions 1:1:1 and *x *= 25) had CPU time of 81.2 seconds (82.1 elapsed time) and CV-5 had CPU time of 434.6 seconds (438.0 elapsed time). For this setting, 3WS is 5.4 times faster than CV-5. When considering 100 datasets, 3WS had CPU time of 8067.0 seconds (average of 80.7 per dataset) and CV-5 had CPU time of 43219.4 seconds (average of 431.2 per dataset). This represents a substantial gain in computational efficiency.

### Real Data Analysis

The results of the real data analysis are shown in Tables 6 and 7. The final model from the MDR analysis, based on maximum cross-validation consistency and maximal testing/prediction accuracy is highlighted in bold. The immunogenetics data was re-evaluated with MDR using 3WS, with *x *= 35, data split 2:2:1, and BIC for post-hoc pruning, and the results are shown in Table 7. The table lists the top models for each level of interaction from the testing test, and the validation accuracy estimated in the validation set for each level of interaction. The overall best model, determined by the pruning was the same two-locus model identified using 5-fold cross-validation, involving SNPs in the IL-2Rβ (16491C > G) and IL-2Rγ (CD132) (4735T > C).

**Table 6:** Results of the 5-fold cross-validation MDR analysis of the HIV pharmacogenomics data.

Number of Loci Evaluated	Polymorphism in Model	Cross Validation Consistency	Prediction Accuracy
1	*IL2RB_6844*	5	55.52

**2**	***CD132_9823, IL2RB_6844***	**5**	**57.22**

3	*IL2_9511, CD132_9823, IL2RB_6844*	2	54.27

4	*IL2_4663, CD132_9823, IL2RB_6844, IL15_87709*	1	52.57

The final model is highlighted in bold.

**Table 7:** Results of the 3WS MDR analysis of the HIV pharmacogenomics data.

Number of Loci Evaluated	Polymorphism in the Best Model in the Testing Set	Validation Accuracy
1	*IL2RB_6844*	56.45

2	*CD132_9823, IL2RB_6844*	57.01

3	*CD132_9823, IL2RB_6844, IL15RA_2990*	58.65

4	*CD132_9823, IL2RB_6844, IL15RA_2990*, *IL15_87710*	62.60

* Best Model after pruning: *CD132_9823, IL2RB_6844*

## Discussion

In the current study, we evaluate the computation time and performance of an alternative internal model validation strategy in the MDR method. We demonstrate that for higher order interactions, the three-way split internal model validation method has similar liberal power to 5-fold cross-validation and clear computational advantages. Additionally, with the application of a post-hoc pruning procedure for final model selection after 3WS, the conservative power of this approach is similar to that of cross-validation. These conclusions from the simulation experiments were validated in a real data application in HIV-1 immunogenetics.

The results of the simulation study confirm some general trends that are common to all association analyses; as both effect size and minor allele frequency increase, power increases, but as size of interaction increases, power decreases. For disease models with a single causative locus, 5-fold cross-validation outperforms the three-way split; but MDR is primarily designed to detect interactions, and many well-established analytical options (both traditional and data-mining methods) are available for single locus models with high power to detect main effects [39]. It should also be noted that in this situation, MDR could not undershoot a single locus model, since that would imply not selecting any loci.

The results of this study have some additional implications for the detection of epistatic effects in genetic association studies. Most notably, 3WS dramatically reduces the computation time of MDR by approximately one fifth from that of CV-5, which has implications for the computationally expensive nature of both the method itself and the permutation testing procedure that is currently utilized to evaluate model significance [7]. The reduction in computing time using the three-way split will make analysis and permutation testing more feasible, facilitating larger genome scans by permitting more loci to be evaluated with MDR. Additionally, by using a three-way split for internal validation, we are able to retain high liberal power as compared to CV-5 and CV-10, or the power to detect interacting causative loci while allowing for false positives. Retaining high liberal power has important implications for studies seeking to identify candidate genes for further investigation by geneticists, as overlooking important genetic factors could be a more critical error than mistakenly including nuisance loci in this situation. By integrating a pruning procedure after the primary 3WS analysis, we are able to reduce the potential problem of false positive findings. In fact, when logistic regression with backward selection is performed using BIC, conservative power equal to that seen with CV-5 is attained. Therefore, by incorporating 3WS with MDR followed by pruning, we are able to achieve performance equivalent to that of cross-validation in a fraction of the time.

Additionally, the cut-off of *x *of the top models sent from the training set to the testing set as well as the relative magnitude of the three data subsets utilized in our simulation study were initially arbitrary, so we investigated the effect of varying these parameters to provide some guidelines for how to select these in practice. We saw that *x *= 25 and the 2:2:1 split maximized performance. Because our datasets consisted of a total of 25 loci, we suggest choosing *x *= total number of loci. A smaller value of *x *could be chosen to reduce computation time. We can generalize from the two proportions yielding the highest liberal power (2:2:1 and 2:1:1) that the three-way split method will yield optimal performance when the validation subset of data is smallest and the magnitude of the training set relative to the testing set is greater than or equal to 1. Computation time is reduced by minimizing the size of the training set, so the 2:2:1 split should minimize computation time while optimizing performance.

In regards to specifics of the pruning procedure, we evaluated several approaches to pruning, including different p-value thresholds and the use of two popular information criteria metrics in conjunction with backward selection for logistic regression. While ultimately these parameter choices are arbitrary, and should be selected based on the specific goals of a particular study (especially in weighting the consequences of false positive versus false negative findings), our simulation study results provide guidance in selecting these parameters. While no pruning will maximize liberal power, our results suggest the use of BIC to maximize conservative power. For application of MDR as a screening tool to prioritize variants to be evaluated in follow-up studies, to minimize false negatives at the expense of an increased false-positive rate, 3WS with no pruning is computationally optimal. For a study involving a strict test of hypotheses, to minimize false positives at the expense of a higher false-negative rate, MDR with 3WS pruned by backward selection using BIC is equivalent to MDR with CV-5, but computationally superior. And MDR with 3WS pruned back by backward selection using a p-value threshold between 0.01 and 0.001 will optimize the balance between the two error rates. Additionally, it should be noted that we considered pruning only under the framework of logistic regression, and that other pruning strategies are possible, and potentially preferable. For instance, the final model identified through logistic regression with backward selection may not be consistent with the high-risk/low-risk MDR model. Some of the advantages of logistic regression are that it is widely available, easy to implement, and well recognized in many fields, but these advantages do not imply or guarantee that logistic regression provides the optimal pruning strategy.

The guidelines for the use of 3WS are demonstrated in the real data analysis of HIV-1 data using a 2:2:1 split, *x *= 35 (the total number of SNPs in the dataset), and BIC for pruning. The results of the 3WS analysis indicate a two-locus epistatic model that predicts immune recovery. This model is the same as the best model identified with 5-fold cross-validation.

While this study is informative in illustrating the powerful potential the three-way split may have in genetic screening for epistasis, it represents a first step in characterizing the utility of the internal validation technique. Because the 3WS before pruning tends to select a model as large as possible, there is a loss in conservative power due to the increased number of false positives. The three-way split could be incorporated into a procedure similar to cross-validation, where the three-way split is performed multiple times and a final model is selected across the multiple three-way splits to achieve more precise prediction estimates. Additionally, the pruning procedure we provide based on logistic regression is just one possibility and other procedures to refine the final model by pruning back false positives could also be considered. Future studies should evaluate additional implementations of internal model validation measures into the MDR algorithm, for instance nested cross-validation, and continue to optimize parameters involved in this process. In particular, preliminary results suggest that the use of a 2WS may have similar performance to 3WS, and could marginally reduce computation time further. While these results are specific for the MDR method, many data-mining approaches rely on cross-validation for internal model validation. Based on the results of this study, the 3WS approach should be evaluated for its potential application to other data-mining methods.

## Conclusions

In the current study we show through simulation that for epistatic models of disease risk, the three-way split internal model validation method has similar power to 5-fold cross-validation to detect the true causative loci while allowing for false positive loci for the MDR approach, and similar power to detect the true causative loci without false positives when pruning is incorporated. Additionally, we show that the computation time required for this procedure is five times less than 5-fold cross-validation. This sizable computational efficiency gain with the maintenance of performance demonstrates the utility of MDR with a three-way split as a screening tool for candidate gene studies.

The incorporation of the three-way split into the MDR method rather than cross-validation in genetic association studies may be fruitful, however, we must consider the trade-off between conservative and liberal power. Researchers will need to decide which type of error is more detrimental based on their research goals, and adjust the pruning procedure to match these goals, balancing false positive and false negative findings. Nevertheless, replication in a separate independent dataset will remain the ideal in assessing the validity of the reported model.

## Competing interests

The authors declare that they have no competing interests.

## Authors' contributions

SJW helped to design the study, performed the simulation and statistical analysis, and drafted the manuscript. AJS performed the parameter sweep and pruning analyses and drafted text and tables for the manuscript. AAM conceived of the study, assisted in its design, and also drafted the manuscript. All authors read and approved the final manuscript.

## Supplementary Material

Additional file 1 **Supplemental Table S1**. This file contains all models considered by combinations of size, minor allele frequency, heritability, and odds ratio. Click here for file

Additional file 2 **Supplemental Table S2**. This file contains all conservative and liberal power results for the comparisons of CV-5, CV-10, and 3WS. Click here for file

Additional file 3 **Supplemental Table S3**. This file contains conservative and liberal power results for comparisons of 3WS, CV-5, and CV-10 for data simulated with 25 or 100 total loci. Click here for file

Additional file 4 **Supplemental Table S4**. This file contains conservative and liberal power results for comparisons between 3WS and 2WS. Click here for file

Additional file 5 **Supplemental Table S5**. This file contains 3WS conservative and liberal power results for various *x *and data proportion parameters in two-locus models. Click here for file

Additional file 6 **Supplemental Table S6**. This file contains additional results for the comparison of 3WS *x *and data proportion parameters in two-locus models, including (a) p-values, (b) power estimates by *x*, (c) power estimates by data proportion, and (d) pair wise contrasts. Click here for file

Additional file 7 **Supplemental Table S7**. This file contains conservative and liberal power for comparison of 3WS data proportion parameters in two- and three-locus models. Click here for file

Additional file 8 **Supplemental Table S8**. This file contains additional results for the comparison of 3WS data proportion parameters in two- and three-locus models, including (a) p-values, (b) power estimates by data proportion, and (c) pair wise contrasts. Click here for file

Additional file 9 **Supplemental Table S9**. This file contains conservative and liberal power results for comparison of pruning methods for 3WS in two- and three-locus models. Click here for file

Additional file 10 **Supplemental Table S10**. This file contains additional results for the comparison of pruning methods for 3WS in two- and three-locus models, including (a) p-values, (b) power estimates by selection criteria, and (c) pair wise contrasts. Click here for file

## References

[B1] Moore JH The ubiquitous nature of epistasis in determining susceptibility to common human diseases Hum Hered 2003 56 1-3 73 82 10.1159/000073735 14614241

[B2] Moore JH A global view of epistasis Nat Genet 2005 37 1 13 14 10.1038/ng0105-13 15624016

[B3] Thornton-Wells TA Moore JH Haines JL Genetics, statistics and human disease: analytical retooling for complexity Trends Genet 2004 20 12 640 647 10.1016/j.tig.2004.09.007 15522460

[B4] Moore JH Ritchie MD STUDENTJAMA. The challenges of whole-genome approaches to common diseases JAMA 2004 291 13 1642 1643 10.1001/jama.291.13.1642 15069055

[B5] Moore JH Williams SM New strategies for identifying gene-gene interactions in hypertension Ann Med 2002 34 2 88 95 10.1080/07853890252953473 12108579

[B6] Moore JH Gilbert JC Tsai CT Chiang FT Holden T Barney N White BC A flexible computational framework for detecting, characterizing, and interpreting statistical patterns of epistasis in genetic studies of human disease susceptibility Journal of Theoretical Biology 2006 241 2 252 261 10.1016/j.jtbi.2005.11.036 16457852

[B7] Ritchie MD Hahn LW Roodi N Bailey LR Dupont WD Parl FF Moore JH Multifactor-dimensionality reduction reveals high-order interactions among estrogen-metabolism genes in sporadic breast cancer Am J Hum Genet 2001 69 1 138 147 10.1086/321276 11404819 PMC1226028

[B8] Ritchie MD Hahn LW Moore JH Power of multifactor dimensionality reduction for detecting gene-gene interactions in the presence of genotyping error, missing data, phenocopy, and genetic heterogeneity Genet Epidemiol 2003 24 2 150 157 10.1002/gepi.10218 12548676

[B9] Motsinger A Hahn LW Dudek SM Ryckman KK Ritchie MD Keijzer M Alternative cross-over strategies and selection techniques for Grammatical Evolution Optimized Neural Networks Genetic and Evolutionary Computation Conference 2006 Association for Computing Machinery Press 947 949 20634918 10.1145/1143997.1144163 PMC2903763

[B10] Namkung J Kim K Yi S Chung W Kwon MS Park T New evaluation measures for multifactor dimensionality reduction classifiers in gene-gene interaction analysis Bioinformatics 2009 25 3 338 345 10.1093/bioinformatics/btn629 19164302

[B11] Lou XY Chen GB Yan L Ma JZ Zhu J Elston RC Li MD A generalized combinatorial approach for detecting gene-by-gene and gene-by-environment interactions with application to nicotine dependence Am J Hum Genet 2007 80 6 1125 1137 10.1086/518312 17503330 PMC1867100

[B12] Gui J Moore JH Andrew AS Robust Multifactor Dimensionality Reduction Method for Detecting Gene-Gene Interaction in Bladder Cancer Genetic Epidemiology 2008 32 7 8417654608

[B13] Chung YJ Lee SY Elston RC Park T Odds ratio based multifactor-dimensionality reduction method for detecting gene-gene interactions Bioinformatics 2007 23 1 71 76 10.1093/bioinformatics/btl557 17092990

[B14] Bush WS Dudek SM Ritchie MD Parallel multifactor dimensionality reduction: a tool for the large-scale analysis of gene-gene interactions Bioinformatics 2006 22 17 2173 2174 10.1093/bioinformatics/btl347 16809395 PMC4939609

[B15] Martin ER Ritchie MD Hahn L Kang S Moore JH A novel method to identify gene-gene effects in nuclear families: the MDR-PDT Genet Epidemiol 2006 30 2 111 123 10.1002/gepi.20128 16374833

[B16] Motsinger-Reif AA Wood SJ Oberoi S Reif DM Epistasis List: A Curated Database of Gene-Gene and Gene-Environment Interactions in Human Genetics ASHG 2008 Philadelphia, PA

[B17] Edwards TL Dudek SM Ritchie MD Resolving the power of multifactor dimensionality reduction in the presence of many noise variables or genetic heterogeneity Genet Epidemiol 2007 31 6 69

[B18] Motsinger AA Ritchie MD The effect of reduction in cross-validation intervals on the performance of multifactor dimensionality reduction Genet Epidemiol 2006 30 6 546 555 10.1002/gepi.20166 16800004

[B19] Motsinger-Reif AA Reif DM Fanelli TJ Ritchie MD A comparison of analytical methods for genetic association studies Genet Epidemiol 2009 10.1002/gepi.20345 18561203

[B20] Velez DR White BC Motsinger AA Bush WS Ritchie MD Williams SM Moore JH A balanced accuracy function for epistasis modeling in imbalanced datasets using multifactor dimensionality reduction Genet Epidemiol 2007 31 4 306 315 10.1002/gepi.20211 17323372

[B21] Motsinger AA Brassat D Caillier SJ Erlich HA Walker K Steiner LL Barcellos LF Pericak-Vance MA Schmidt S Gregory S Hauser SL Haines JL Oksenberg JR Ritchie MD Complex gene-gene interactions in multiple sclerosis: a multifactorial approach reveals associations with inflammatory genes Neurogenetics 2007 8 1 11 20 10.1007/s10048-006-0058-9 17024427

[B22] Brassat D Motsinger AA Caillier SJ Erlich HA Walker K Steiner LL Cree BA Barcellos LF Pericak-Vance MA Schmidt S Gregory S Hauser SL Haines JL Oksenberg JR Ritchie MD Multifactor dimensionality reduction reveals gene-gene interactions associated with multiple sclerosis susceptibility in African Americans Genes Immun 2006 7 4 310 315 10.1038/sj.gene.6364299 16625214 PMC4339061

[B23] Edwards TL Wang X Chen Q Wormly B Riley B O'Neill FA Walsh D Ritchie MD Kendler KS Chen X Interaction between interleukin 3 and dystrobrevin-binding protein 1 in schizophrenia Schizophr Res 2008 106 2-3 208 217 10.1016/j.schres.2008.07.022 18804346 PMC2746913

[B24] Nordgard SH Ritchie MD Jensrud SD Motsinger AA Alnaes GI Lemmon G Berg M Geisler S Moore JH Lonning PE Borresen-Dale AL Kristensen VN ABCB1 and GST polymorphisms associated with TP53 status in breast cancer Pharmacogenet Genomics 2007 17 2 127 136 10.1097/FPC.0b013e328011abaa 17301692

[B25] Witten FFE Data Mining: Practical Machine Learning Tools and Techniques (Second Edition) 2005 Morgan Kaufmann

[B26] Ioannidis JP Ntzani EE Trikalinos TA Contopoulos-Ioannidis DG Replication validity of genetic association studies Nat Genet 2001 29 3 306 309 10.1038/ng749 11600885

[B27] Hastie TJ Tibshirani RJ Friedman JH The elements of statistical learning 2001 Basel: Springer Verlag

[B28] Hahn LW Ritchie MD Moore JH Multifactor dimensionality reduction software for detecting gene-gene and gene-environment interactions Bioinformatics 2003 19 3 376 382 10.1093/bioinformatics/btf869 12584123

[B29] Culverhouse R Suarez BK Lin J Reich T A perspective on epistasis: limits of models displaying no main effect Am J Hum Genet 2002 70 2 461 471 10.1086/338759 11791213 PMC384920

[B30] Gatz M Pedersen NL Berg S Johansson B Johansson K Mortimer JA Posner SF Viitanen M Winblad B Ahlbom A Heritability for Alzheimer's disease: The study of dementia in Swedish twins Journals of Gerontology Series a-Biological Sciences and Medical Sciences 1997 52 2 M117 M125 10.1093/gerona/52a.2.m117 9060980

[B31] Li W Reich J A complete enumeration and classification of two-locus disease models Hum Hered 2000 50 6 334 349 10.1159/000022939 10899752

[B32] Frankel WN Schork NJ Who's afraid of epistasis? Nat Genet 1996 14 4 371 373 10.1038/ng1296-371 8944011

[B33] Moore JH Hahn L Ritchie MD Thornton-Wells TA White BC Routine discovery of high order epistasis models for simulation studies in human genetics Applied Soft Computing 2004 4 7 10.1016/j.asoc.2003.08.003 PMC2952957 20948983

[B34] Dudek SM Motsinger AA Velez DR Williams SM Ritchie MD Data simulation software for whole-genome association and other studies in human genetics Pac Symp Biocomput 2006 499 510 full_text 17094264

[B35] Haas DW Geraghty DE Andersen J Mar J Motsinger AA D'Aquila RT Unutmaz D Benson CA Ritchie MD Landay A Immunogenetics of CD4 lymphocyte count recovery during antiretroviral therapy: An AIDS Clinical Trials Group study J Infect Dis 2006 194 8 1098 1107 10.1086/507313 16991084

[B36] Davidian M Giltinian DM Nonlinear Models for Repeated Measurement Data 1995 Chapman and Hall7618257

[B37] Hedeker D Gibbons R Longitudinal Data Analysis 2006 Hoboken, New Jersey: John Wiley and Sons, Inc

[B38] R Development Core Team R: A language and environment for statistical computing 2005 R Foundation for Statistical Computing. Vienna, Austria http://www.R-project.org ISBN 3-900051-07-0

[B39] Motsinger-Reif AA Reif DM Fanelli TJ Ritchie MD A comparison of analytical methods for genetic association studies Genet Epidemiol 2008 32 8 767 778 10.1002/gepi.20345 18561203

